# Urolithin a attenuates IL-1β-induced inflammatory responses and cartilage degradation via inhibiting the MAPK/NF-κB signaling pathways in rat articular chondrocytes

**DOI:** 10.1186/s12950-020-00242-8

**Published:** 2020-03-24

**Authors:** Sheng-long Ding, Zhi-ying Pang, Xue-mei Chen, Zheng Li, Xin-xin Liu, Qi-lin Zhai, Jun-ming Huang, Zhi-yong Ruan

**Affiliations:** 1 Department of Orthopaedic Surgery, Qingpu Branch of Zhongshan Hospital, Fudan University, 1158 Gong Yuan Dong Road, Qingpu District, Shanghai, 201700 China; 2 Department of Orthopaedic Surgery, Zhongshan Hospital, Fudan University, 180 Feng Lin Road, Xuhui District, Shanghai, 200032 China; 3 Department of Anesthesiology, Shanghai JiaoTong University Affiliated Shanghai Sixth People’s Hospital, Shanghai, 200233 China

**Keywords:** Urolithin a, Osteoarthritis, NF-κB, MAPK, Inflammation

## Abstract

**Background:**

Osteoarthritis (OA) is characterized by inflammation and extracellular matrix (ECM) degradation and is one of the most common chronic degenerative joint diseases that causes pain and disability in adults. Urolithin A (UA) has been widely reported for its anti-inflammatory properties in several chronic diseases. However, the effects of UA on OA remain unclear. The aim of the current study was to investigate the anti-inflammatory effects and mechanism of UA in interleukin-1β (IL-1β)-induced chondrocytes.

**Results:**

No marked UA cytotoxicity was noted, and UA protected cartilage from damage following IL-1β stimulation in micromasses. Moreover, UA promoted the expression of anabolic factors including Sox-9, Collagen II, and Aggrecan while inhibiting the expression of catabolic factors such as matrix metalloproteinases (MMPs) and a disintegrin and metalloproteinase with thrombospondin motifs 4 (ADAMTS-4) in rat chondrocytes. Protective effects of UA were also observed in ex vivo organ culture of articular cartilage. Mechanistically, IL-1β significantly activated and upregulated the expression of p-ERK 1/2, p-JNK, p-P38, and p-P65, while UA protected chondrocytes against IL-1β-induced injury by activating the mitogen-activated kinase (MAPK)/nuclear factor-κB (NF-κB) signaling pathways.

**Conclusion:**

Our results provide the evidence that UA could attenuate IL-1β-induced cell injury in chondrocytes via its anti-inflammatory action. UA may be a promising therapeutic agent in the treatment of OA.

## Introduction

Osteoarthritis (OA) is one of the most common forms of chronic degenerative joint disease and affects tens of millions of people around the world [1]. The main characteristic features observed in OA include progressive loss and destruction of articular cartilage, thickening of the subchondral bone, osteophyte formation, and synovial inflammation Multiple factors contribute to the initiation and progression of OA, such as aging, heredity, obesity, abnormal metabolism, joint injury, osteoporosis, and joint malformation [2, 3].

At the cellular and molecular levels, inflammation and inflammatory mediators play crucial roles in initiating and accelerating OA development [4, 5]. A growing body of evidence suggests that interleukin-1β (IL-1β), tumor necrosis factor-alpha (TNF-α), and IL-6 are found in OA cartilage [6]. Among these inflammatory cytokines, the effect of IL-1β was widely explored because of its vital role in inflammatory responses. The pro-inflammatory cytokine IL-1β is a master regulator of inflammation that has been reported to directly participate in the generation of multiple inflammatory mediators [3]. When chondrocytes are stimulated by IL-1β, they produce metalloproteinases (MMPs), a metalloproteinase with a thrombospondin type 1 motifs (ADAMTS), and some inflammation-associated proteins including inducible nitric oxide synthase (iNOS) and cyclooxygenase-2 (COX-2), which trigger the alteration of cartilage from the normal homeostatic state toward a catabolic state and eventually leads to extracellular matrix (ECM) degradation [7, 8]. Therefore, targeting IL-1β-induced catabolic metabolism and inflammatory responses may be an effective strategy to delay OA progression.

Urolithin A (UA) is metabolized by intestinal microbiota from Ellagitannins (ETs) and Ellagic acid (EA) in the gut [9, 10]. According to previous studies, ETs and EA may inhibit the inflammatory response. Dietary consumption of EA-rich food has been demonstrated to suppress inflammatory cytokine release in the brains of Alzheimer’s disease mice [11]. Similarly, EA protects against cisplatin-induced kidney nephrotoxicity by inhibiting renal inflammation and apoptosis [12]. Nevertheless, EA is poorly absorbed and quickly eliminated, and the biological activity of EA is controversial. Interestingly, recent published studies have described the biological effects of UA, including anti-proliferation in cancer, anti-inflammation, anti-oxidant activity, improved lipid metabolism [13–15]. UA inhibits the catabolic effect of TNF-α on nucleus pulposus cells and alleviates intervertebral disc degeneration in vivo [16]. Moreover, UA can protect skeletal muscle against acute inflammation in vitro and in vivo [17]. Mechanistically, UA could significantly inhibit the activation of NF-κB induced by IL-1β in colon fibroblasts [18]. Meanwhile, Fu et al. investigated the anti-inflammatory effect of UA in human OA and revealed the underlying mechanism by blockage of PI3K/Akt/NF-κB pathway [19]. Although the potential anti-inflammatory role of UA has been extensively investigated, there is limited knowledge whether UA has other potential therapeutic targets to attenuate the pathogenesis of OA. In this study, we investigated the anti-inflammatory role of UA by attenuating IL-1β-induced degradation of Collagen II and Aggrecan and by reducing the production of inflammatory mediators via the ERK, JNK, P38, and NF-κB pathways in rat chondrocytes.

## Materials and methods

### Antibodies and reagents

Urolithin A (CAS No. 1143-70-0) was bought from Cayman Chemical (Ann Arbor, MI, USA). Recombinant rat IL-1β (400-01B) was purchased from (Peprotech, Suzhou, China). Fetal bovine serum (FBS) was provided by Gibco Life Technologies (Grand Island, NY, USA). Antibodies against antiphospho-ERK (#4370), ERK (#9102), antiphospho-JNK (#4668), JNK (#9252), antiphospho-p38 (#4511), p38 (#8690), antiphospho-p65 (#3033), p65 (#8242), COX2 (#12282), iNOS (#13120) Sox-9 (#82630) were bought from Cell Signaling Technology (Danvers, MA, USA). Antibodies against MMP3 (ab52915), MMP13 (ab39012), Collagen II (ab34712), aggrecan (ab36861) were purchased from Abcam (Cambridge, UK). Horseradish peroxidase (HRP)-conjugated glyceraldehyde 3-phosphate dehydrogenase (GAPDH) (ET1702–66) and HRP-linked goat anti-rabbit (HA1001) were supplied by Huabio (Hangzhou, China). Phalloidin was provided by Beyotime (Shanghai, China). Cy3- conjugated goat anti-rabbit secondary antibody (BA1032) and 4′,6-diamidino-2-phenylindole (DAPI) (AR1177) were purchased from Boster (Wuhan, China). Other reagents were of the highest commercial grade and were purchased from Sigma Chemical (St. Louis, MO, USA).

### Cell culture

Primary chondrocytes were obtained from knee joints cartilage of 2-week-old Sprague Dawley rats. The detailed procedure was performed according to a previously described method [20]. Briefly, cartilage of the knee joint was isolated and cut into pieces and then incubated in 0.5% trypsin-EDTA (containing 0.5 g/L of trypsin (1:250) and 0.2 g/L EDTA•4 Na in 0.85% saline solution) for 30 min and subsequently 0.2% collagenase for 24 h at 37 °C. The chondrocytes were collected and cultured in Dulbecco’s minimum essential medium: F12 medium containing 10% FBS with humid air with 5% CO_2_ at 37 °C. Cells were trypsinized with 0.5% trypsin-EDTA and passaged at a ratio of 1:3 when cell density reaches 75%, and the medium was changed every 2 days. Chondrocytes at passage 3 were utilized in the subsequent experiments.

### Cell viability assay

The Cell Counting Kit-8 (CCK8, Dojindo, Japan) was utilized to analyze cell viability. Firstly, chondrocytes were seeded in 96-well plates at a density of 1 × 10^4^ cells/well. After 24 h of adhesion, cells were then treated with IL-1β alone or with UA at different concentrations. Cell viability was carried out after cultivating for 1, 3, and 7 days. In brief, 10 μl CCK-8 solution dissolved in 100 μl culture medium was added into each well and then incubated in the dark at 37 °C for 1.5 h. The absorbance of the solution was recorded at 450 nm using a plate reader (BioTek, Winooski, VT, USA).

### Micromass culture

All procedures were performed as previously described [21, 22]. Briefly, the chondrocytes were suspended in medium with 10% FBS, 0.25% penicillin-streptomycin, and 0.25% L-glutamine, and plated at a density of 2.5 × 10^5^ cells/10 μl in 24-well plates. Four hours later, the medium was added into the plate with IL-1β alone or with IL-1β with UA for 2 days. Then the micromasses were stained with Alcian Blue.

### Western blotting analysis

Chondrocytes were cultured in a sterile six-well plates at 37 °C with 5% CO_2_. After reaching 80% density, the cells were exposed to IL-1β alone or with UA. The total proteins were obtained from stimulated or control chondrocytes using radioimmunoprecipitation assay lysis buffer containing 1% proteinase inhibitor and 1% phosphatase inhibitor cocktail for 30 min on ice at the indicated time points. Protein concentrations were measured using BCA protein assay kits (Boster). Then, 40 μg of protein was separated on 12% sodium dodecyl sulfate-polyacrylamide gels and transferred to polyvinylidene fluoride membranes (Millipore, Burlington, MA, USA), blocked with 5% bovine serum albumin (BSA) in Tris-buffered saline with 0.1% Tween-20 (TBS-T) and incubated with primary antibody (2% BSA in TBS-T) overnight at 4 °C. Subsequently, the membrane was washed with TBS-T and incubated with the corresponding secondary antibodies for 2 h at room temperature. Finally, the protein bands were visualized with western ECL Substrate Kits (Yseasen, Shanghai, China) on a Tanon imaging system, and grayscale images were analyzed with ImageJ (National Institutes of Health, Bethesda, MD, USA)/Olympus (Tokyo, Japan) software.

### Total RNA extraction and quantitative real-time RT-PCR

Total RNA was extracted by a total RNA extraction kit (Omega Bio-tek, Norcross, GA, USA) from chondrocytes exposed to IL-1β alone or with UA in accordance with the manufacturer’s instructions. RNA purity and concentration were determined by a spectrophotometer (Thermo Fisher Scientific, Waltham, MA, USA). Complementary DNA (cDNA) was synthesized from total RNA and amplified with SYBR Green Master Mix in an ABI PRISM 7500 PCR Sequence Detection System (Applied Biosystems, Foster City, CA, USA) according to following condition: 30 s of denaturation followed by 40 cycles of 94 °C for 5 s and 60 °C for 35 s. The melting curve was generated to test for primer dimer formation and false priming for each reaction. Relative expressions of gene-specific products were analyzed using the comparative Ct (2^−ΔΔCt^) method and normalized to the reference gene GAPDH. The sequences of primers constructed were as follows: ADAMTS4: forward (CCGTTCCGCTCCTGTAACACTAAG), reverse (AGGTCGGTTCGGTGGTTGTAGG); MMP9: forward (CTACACGGAGCATGGCAACGG), reverse (TGGTGCAGGCAGAGTAGGAGTG); Col2a1: forward (ACGCTCAAGTCGCTGAACAACC), reverse (ATCCAGTAGTCTCCGCTCTTCCAC); GAPDH: forward (GACAATTTTGGCATCGTGGA), reverse (ATGCAGGGATGATGTTCTGG).

### Immunofluorescence

Chondrocytes were plated in 12-well plates. When the density reached 80%, the cells were stimulated with IL-1β alone or with UA. Next, cells were fixed in 4% paraformaldehyde for 15 min at room temperature. Subsequently, the cells were permeabilized in phosphate-buffered saline (PBS) containing 0.3% Triton X-100 for 15 min and then blocked with 5% BSA for 30 min. Cells were then incubated with anti-P65 (1:200 dilution) in a humid chamber overnight at 4 °C. The next day, the plates were washed three times with PBS and then incubated with Cy3-conjugated goat antirabbit secondary antibody (1:100 dilution) at 37 °C for 1 h in the dark. Finally, cells were stained with phalloidin and DAPI. Images were acquired using an inverted fluorescence microscope (Olympus) with identical acquisition settings, and the results were statistically analyzed using ImageJ software.

### Ex vivo organ culture of rat articular cartilage

All experimental protocols were approved by the Committee of Ethics of Animal Experiments at Zhongshan Hospital, Fudan University, China. Cartilage explants were obtained from the knee joints of 4-week-old Sprague Dawley rats that were group-housed at 20 ± 5 °C (55 ± 5% humidity) on a 12-h light/dark cycle with free access to standard chow and water. The detailed procedure was described in a published protocol [23]. Initially, the explants were cultured in medium containing 10% FBS at 37 °C with 5% CO_2_ for 2 days. Then, the explants were cultured in medium (10% FBS and 0.25% penicillin-streptomycin) containing IL-1β and/or IL-1β with UA for 3 additional days. Next, explants were collected and fixed in 4% paraformaldehyde, sectioned at 6 μm, and stained with hematoxylin and eosin (H&E), Safranine O-Fast Green (S-O Fast green), or Alcian Blue, then we used the Osteoarthritis Research Society International (OARSI) scoring system with double blindness as described previously to evaluate the destruction of articular cartilage, scoring including the matrix staining, cartilage tissue structure, chondrocyte clusters, and surface integrity [24, 25]. Further, Collagen type II and aggrecan were analyzed by immunochemistry and The percentages of Collagen II, Aggrecan positive cells in each section were quantified by Image Pro Plus. All stained sections were imaged using an upright microscope (Olympus).

### Statistical analysis

The experiments were performed at least three times. All data are presented as mean ± standard deviation (SD). Statistical analyses were performed using GraphPad Prism software (GraphPad Inc., San Diego, CA, USA) and SPSS 18.0 (IBM, Armonk, NY, USA). For differences among treatments, Student’s *t*-tests were used for the comparisons between two groups, and data involving more than two groups were analyzed by one-way analysis of variance followed by Tukey post hoc tests. *P* values less than 0.05 were considered statistically significant.

## Results

### Cell viability after IL-1β or/and UA treatment

**Fig. 1:**
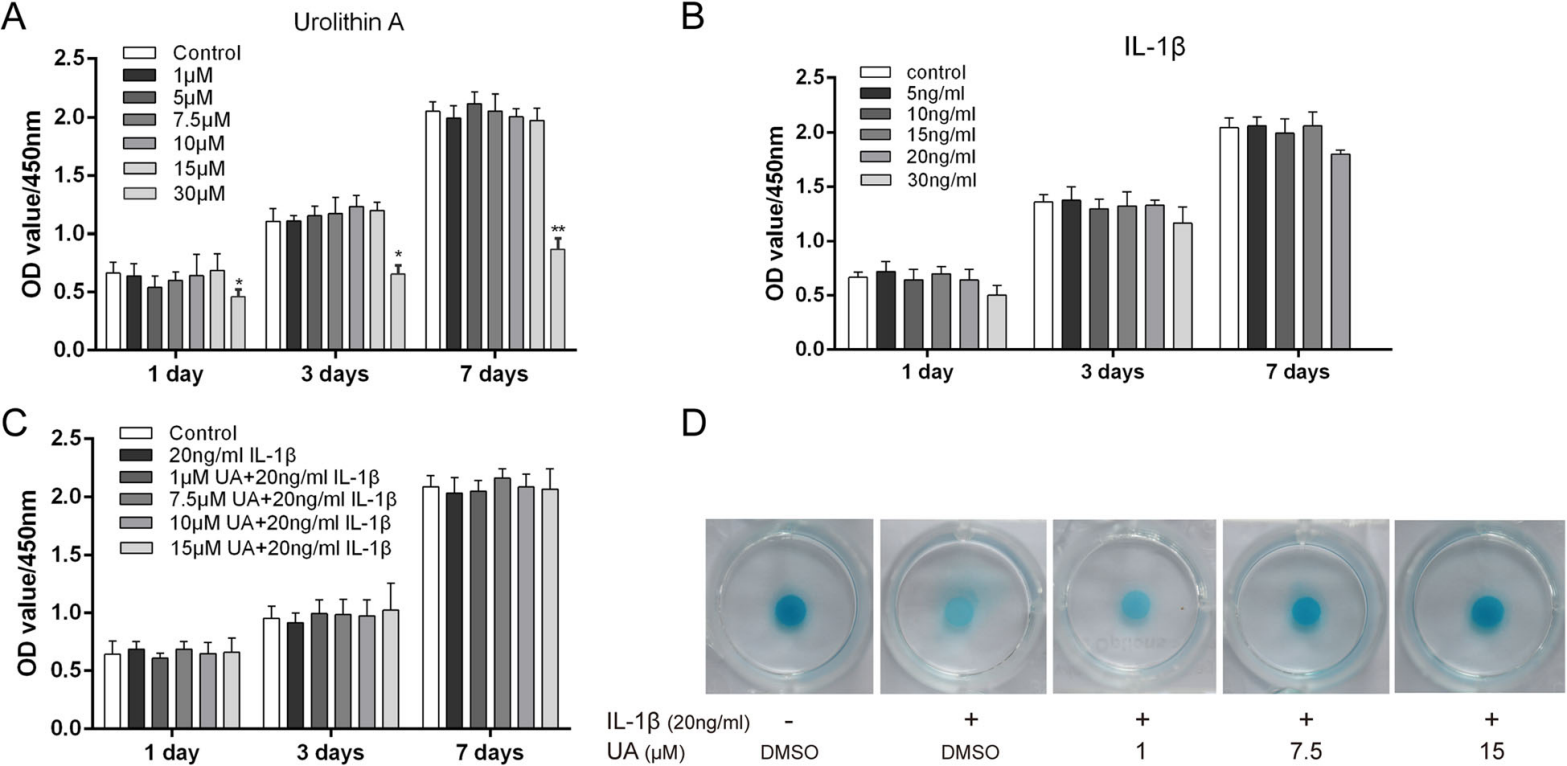
Effect of UA on chondrocyte viability. **a**, **b**, **c** Chondrocytes were treated with various concentrations of UA and/or IL-1β and then analyzed by CCK-8 assay (1, 3, and 7 days). **d** Chondrocytes were treated with 20 ng/ml IL-1β combined with different concentrations of UA (1, 7.5, and 15 μM) for 2 days and then stained with Alcian Blue. Data are presented as mean ± S.D. *n* = 6, *P < 0.05, **P < 0.01 versus Control

First, we examined the potential toxicity of UA on chondrocytes with CCK8 assays. As shown in Fig. 1a, UA had no significant effect on chondrocyte viability and proliferation at concentrations of 1, 5, 7.5, or 15 μM for 1, 3, or 7 days. However, chondrocyte activity decreased by ~ 50% compared with the control group (*P* < 0.05) when the concentration reached 30 μM, indicating that a high concentration of UA may inhibit cell activity. Therefore, we set the maximum concentration of UA to 15 μM (1, 7.5, 15 μM) for subsequent experiments. When chondrocytes were treated with IL-1β for 1, 3, or 7 days, as shown in Fig. 1b and c, there were no significant changes in cell viability with increasing concentrations of IL-1β (< 30 ng/ml) with and without UA (< 15 μM). To investigate whether UA protects against cell damage induced by IL-1β, cartilage micromasses were co-incubated with 20 ng/ml IL-1β and various concentrations of UA from 1 to 15 μM for 2 days and then stained with Alcian Blue. UA markedly ameliorated IL-1β-induced degradation of cartilage matrix in a dose-dependent manner (Fig. 1d). These results suggest that no marked UA cytotoxicity occurred in chondrocytes, and UA partially protected against IL-1β-induced cartilage matrix degradation.

### UA inhibited IL-1β-induced ECM catabolism in chondrocytes

**Fig. 2:**
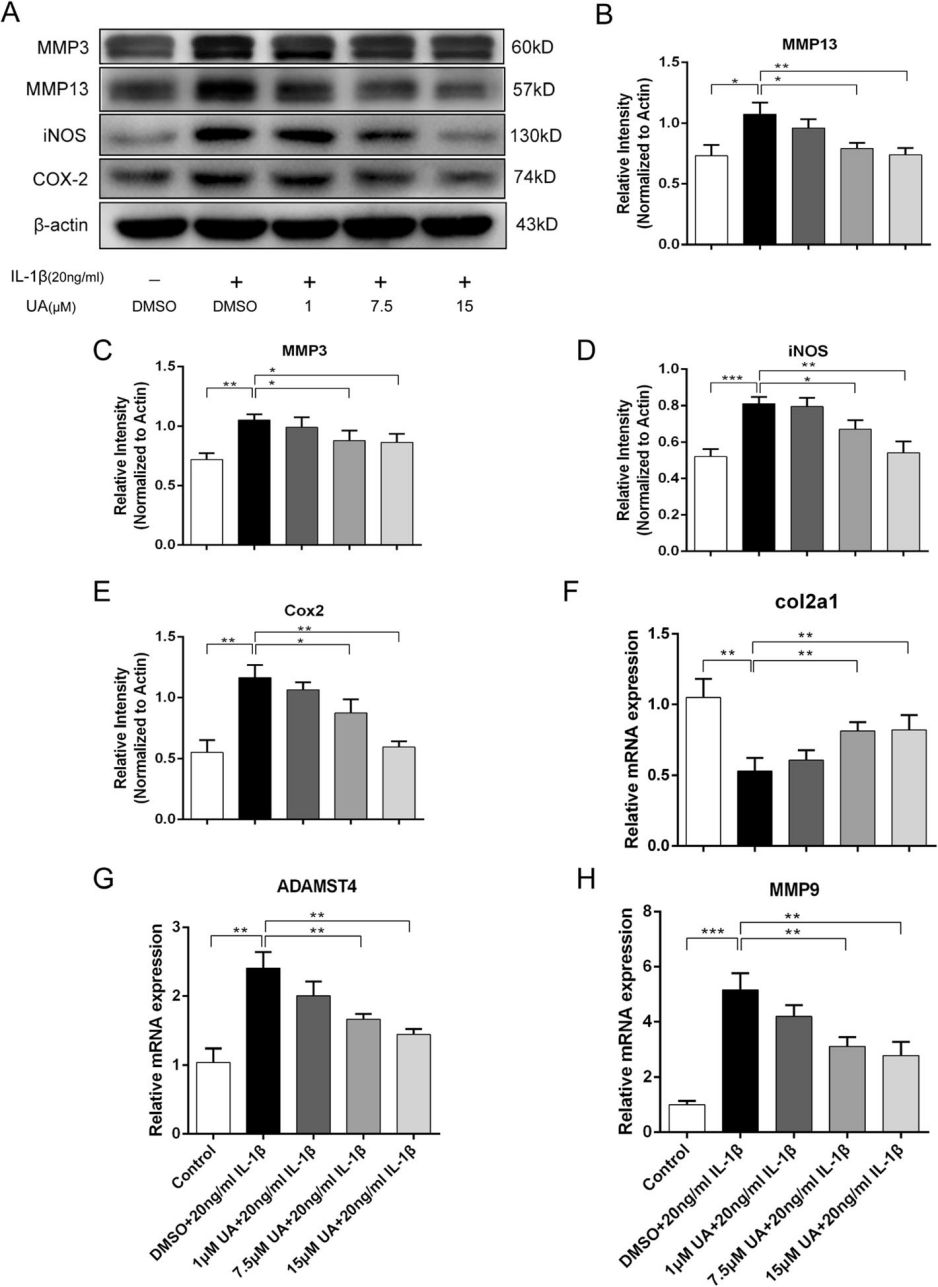
Effect of UA on IL-1β induced expression of iNOS, COX2, and MMPs. Chondrocytes were treated with IL-1β (20 ng/ml) alone or UA (1, 7.5 and 15 μM) in combination with IL-1β (20 ng/ml) for 48 h. **a** Protein expression of iNOS, COX2, MMP3, and MMP13 were determined by western blot. **b**, **c**, **d**, **e** Relative protein expression of iNOS, COX2, MMP3, and MMP13 shown as histograms. **f**, **g**, **h** Relative gene expression of MMP9, ADAMTS4, and Collagen 2a1 were measured by RT-qPCR. Data are presented as mean ± S.D. *n* = 6. *P < 0.05, **P < 0.01, ***P < 0.001 versus the IL-1β group

ECM gene expression levels were detected with RT-qPCR after treatment with various UA concentrations (0, 1, 7.5, 10 μM) for 48 h. MMP9 and ADAMTS4 are matrix-degrading enzymes, and Collagen II could antagonize this effect and promote ECM anabolism. MMPs (MMP3 and MMP13) are catabolic enzymes of Collagen II and Aggrecan. As shown in Fig. 2g and h, MMP9 and ADAMTS4 mRNA expression markedly increased in a dose-dependent manner. UA obviously suppressed the overproduction of MMP9 and ADAMTS4 mRNA induced by IL-1β stimulation. Meanwhile, UA reversed the downregulated gene expression of Collagen II in the IL-1β stimulated condition. However, UA did not affect the expression of these genes at the lowest concentration (1 μM). The effect of UA on IL-1β-induced MMP 3 and MMP13 production were measured by western blot. UA treatment partially reduced protein expression of MMP 3 and MMP13 compared to cells treated with IL-1β alone (Fig. 2a).

### UA prevented IL-1β-induced degradation of Sox-9, collagen II, and Aggrecan

**Fig. 3:**
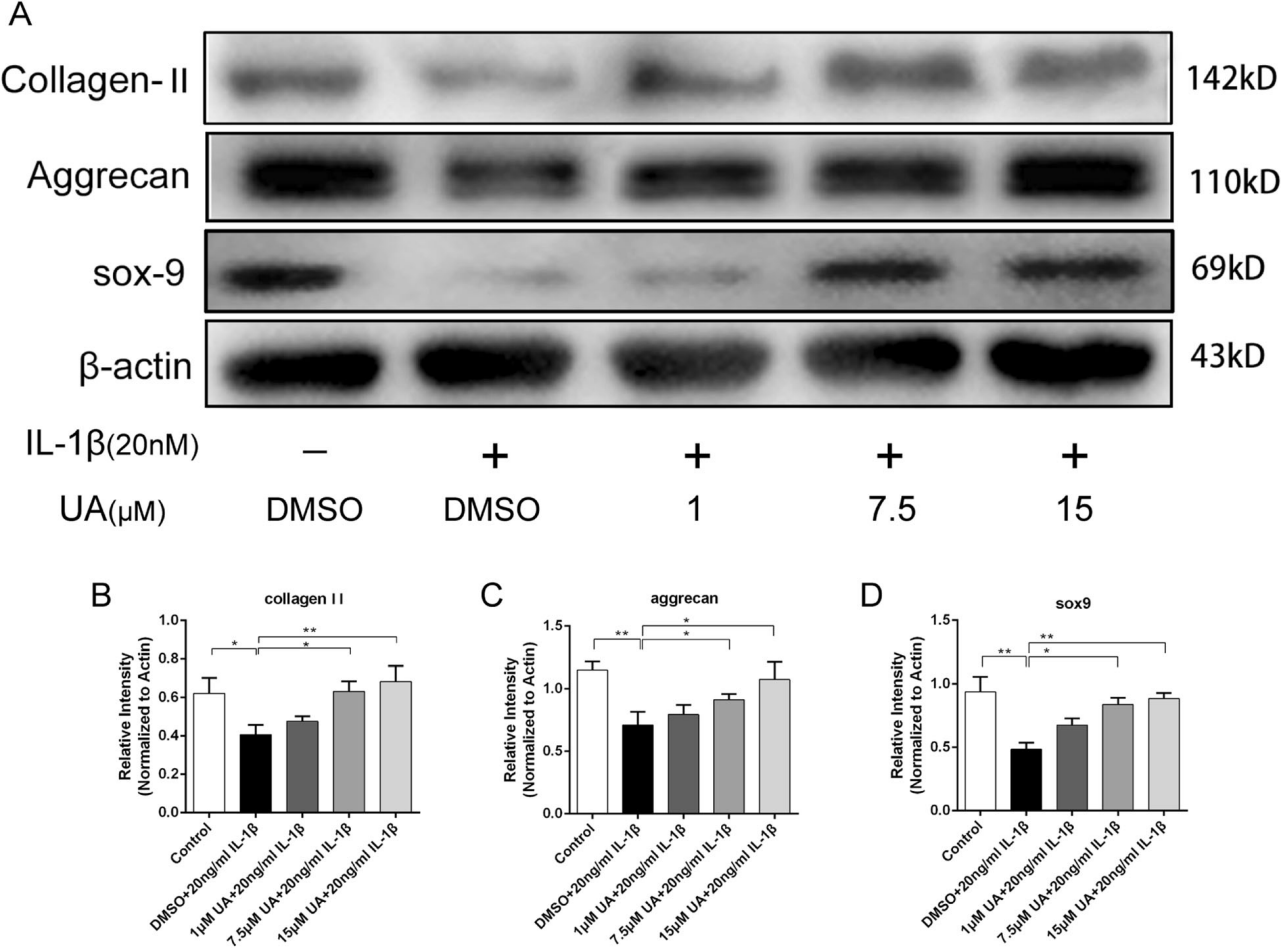
Effect of UA on IL-1β induced degradation of Sox-9, Collagen II, and Aggrecan. Chondrocytes were treated with IL-1β (20 ng/ml) alone or UA (1, 7.5, and 15 μM) in combination with IL-1β (20 ng/ml) for 24 h. **a** Protein expression of Sox-9, Collagen II, and Aggrecan were determined by western blot. **b**, **c**, **d** Relative protein expression of Sox-9, Collagen II, and Aggrecan shown as histograms. Data are presented as mean ± S.D. *n* = 6. *P < 0.05, **P < 0.01, ***P < 0.001 versus the IL-1β group

To evaluate chondrocyte degeneration, we investigated ECM replacement by chondrocytes under IL-1β stimulation with or without UA pretreatment by western blot analysis. Collagen II and Aggrecan are the two main components of cartilage matrix responsible for the anti-compression and shock absorption capabilities of cartilage under mechanical loading. Fig. 3a-c show that IL-1β significantly decreased protein expression of Collagen II (*P* < 0.05) and Aggrecan (*P* < 0.01). However, these alterations were reversed by pretreatment with UA, especially at the highest concentration of 15 μM. The key regulator of Collagen II synthesis is Sox-9, and UA could prevent its degradation induced by IL-1β (*P* < 0.01, Fig. 3a and d). This result was consistent with the RT-qPCR findings.

### UA suppressed IL-1β-induced expression of iNOS and COX2 in chondrocytes

Protein expression levels of iNOS and COX-2 were quantified to examine the extent of IL-1β-induced inflammation and evaluate whether it is attenuated by UA. Chondrocytes were pretreated with different concentrations of UA (1–15 μM) for 2 h and then simulated with or without IL-1β (20 ng/ml) for 48 h. Western blotting was performed to detect protein expression of the inflammatory mediators iNOS and COX2. As shown in Fig. 2a, IL-1β stimulation significantly increased iNOS and COX2 production. However, UA inhibited the excessive production of these mediators. Our results demonstrate that UA co-treatment significantly (*P* < 0.05) and dose dependently decreased the inflammation induced by IL-1β. However, the lowest dose of UA (1 μM) had no protective effects (*P* > 0.05).

### Effect of UA on IL-1β-induced activation of the MAPK pathway

**Fig. 4:**
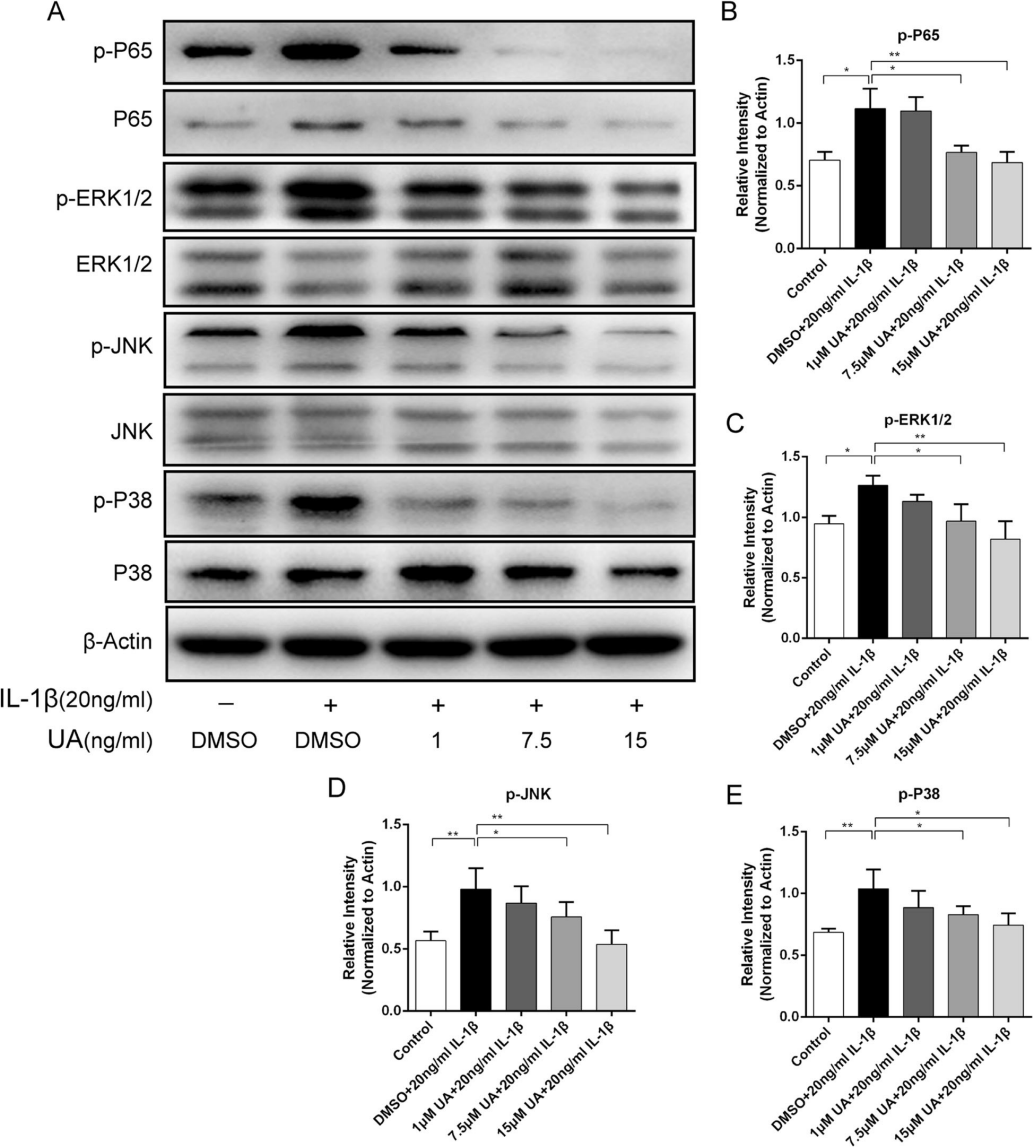
Effect of UA on IL-1β-induced activation of MAPK and NF-κB. Chondrocytes were pretreated with UA (1, 7.5 and 15 μM) for 2 h, followed by co-incubation with 20 ng/ml IL-1β for 30 min. **a** Protein expression of P-ERK, ERK, P-JNK, JNK, P-P38, P38, P-P65, and P65 were determined by western blot. **b**, **c**, **d**, **e** Relative protein expression of P-ERK, P-JNK, P-P38, and P-P65 compared to ERK, JNK, P38, and P65 shown as histograms. Data are presented as mean ± S.D. *n* = 6. *P < 0.05, **P < 0.01, ***P < 0.001 versus the IL-1β group

Previous studies have demonstrated that IL-1β could trigger inflammation by activating the mitogen-activated protein kinase (MAPK) pathway [26]. Specifically, MAPK signaling mediates inflammation responses and cartilage degradation in the pathogenesis of OA. To clarify the mechanism of action underlying UA protection, MAPK activity evaluated using western blot analysis. The phosphorylation levels of ERK, JNK, and p38 were significantly upregulated compared to the control group after treatment with IL-1β for 2 h (*P* < 0.01). Notably, UA could suppress the upregulated phosphorylation of ERK1/2, JNK, and p38 in a concentration-dependent manner (Fig. 4a-d). These results suggest that UA protects chondrocytes against IL-β-induced inflammation injury by inhibiting the phosphorylation of MAPK pathway members.

### UA inhibited IL-1β-mediated activation of the NF-κB pathway

**Fig. 5:**
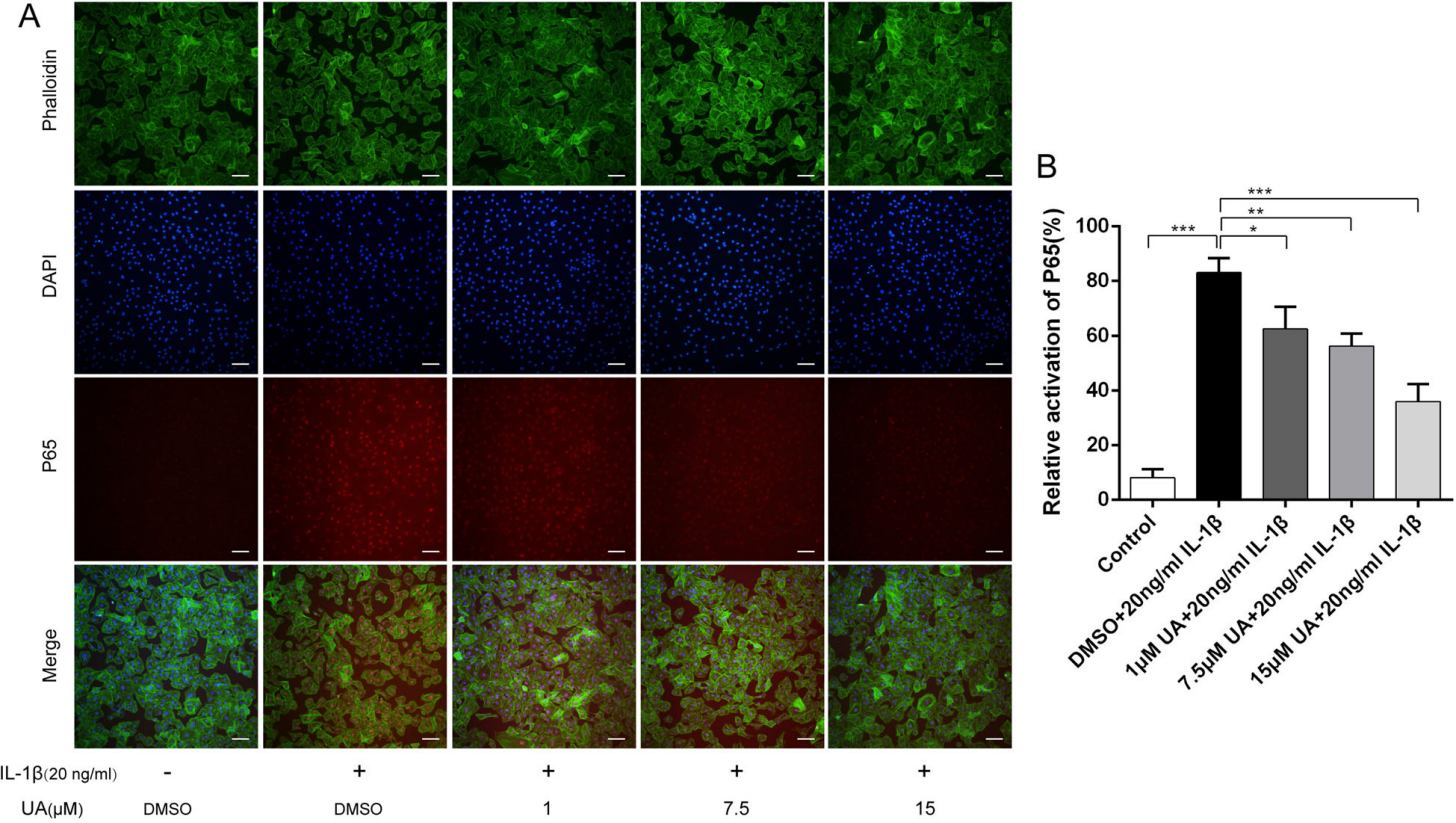
Effect of UA on IL-1β-induced nuclear translocation of P65. Chondrocytes were pretreated with UA (1, 7.5, and 15 μM) for 2 h, followed by co-incubation with 20 ng/ml IL-1β for 30 min and nuclear translocation of NF-κB P65 was detected by immunofluorescence. **a** The nuclear translocation of P65 detected by immunofluorescence (red signal represents P65, original magnification 100×, scale bar: 100 μm). **b** Relative activation of P65 shown as a histogram. Data are presented as mean ± S.D. *n* = 6. *P < 0.05, **P < 0.01, ***P < 0.001 versus the IL-1β group

To further explore the anti-inflammatory mechanism of UA, immunofluorescence and western blot analyses of NF-κB p65 were performed to evaluate the effect of UA on the NF-κB pathway. IL-1β significantly up-regulated p65 phosphorylation (*P* < 0.01). As expected, UA remarkably inhibited IL-1β-induced NF-κB activation in a dose-dependent manner (Fig. 4a and b). However, it is worth noting that phosphorylated p65 was lower than in the control group and the inhibitory effect of UA did not increase at concentrations > 7.5 μM. Immunofluorescence showed that most p65 was present in the cytoplasm in control cells. However, as shown in Fig. 5a and b, IL-1β treatment significantly increased p65 fluorescence intensity, indicating that NF-κB activation induced its nuclear translocation and subsequent transcription of inflammatory mediators. Moreover, chondrocytes treated with 20 ng/ml IL-1β for 2 h exhibited nearly 80% activated p65 was activated, as demonstrated by an ~ 8-fold increase in fluorescence intensity compared to the control group. However, UA pretreatment inhibited p65 translocation into the nucleus (Fig. 5a and b). This observation was consistent with the western blot results. Collectively, these findings suggest that UA protects chondrocytes against IL-β-induced inflammation injury by inhibiting phosphorylation of a member of the NF-κB pathway (p65).

### UA inhibited damage in cartilage explant culture

**Fig. 6:**
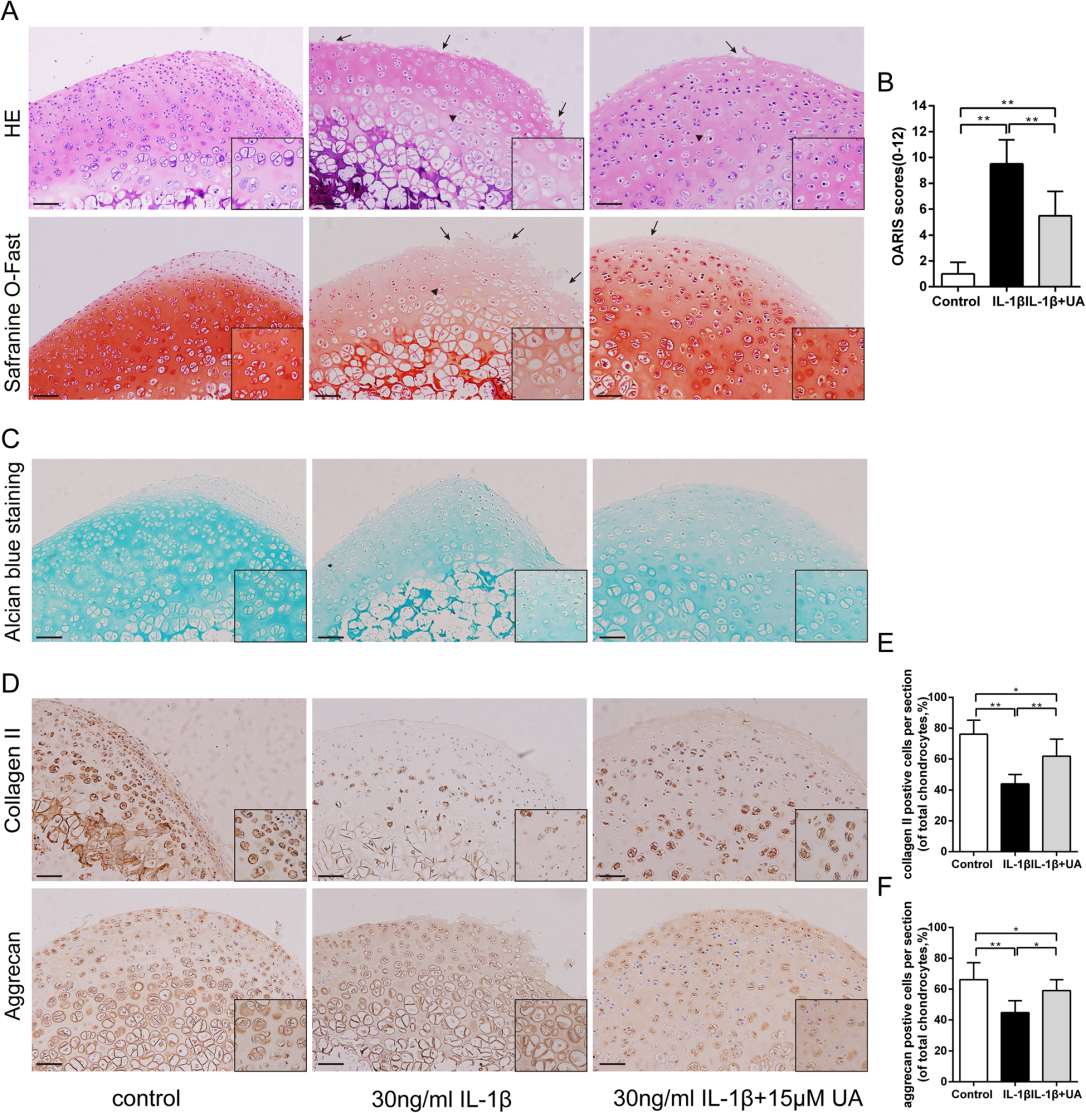
Effect of UA on IL-1β-induced cartilage damage in cartilage explants culture. Rat cartilage explants were exposed to 30 ng/ml IL-1β alone or with 15 μM UA for 3 days. **a**. Representative H&E and S-O Fast Green staining revealed the IL-1β treated group had apparent morphological changes including rough surfaces (black arrow), clustered and disorganized chondrocytes (black triangle), obvious hypocellularity, and loss of Safranin-O staining compared with the control group (scale bar: 20 μm). **b**. Diagram showing the OARSI scores of the cartilage. **c**. Alcian Blue staining for glycosaminoglycan (GAG) distribution (scale bar: 20 μm). **d**. Immunohistochemical staining of Collagen II, Aggrecan expression in the cartilage samples (scale bar: 20 μm). **e** and **f**. The percentages of Collagen II and Aggrecan positive cells in each section were quantified by Image Pro Plus. Three sections were randomly selected for quantification, and original magnification were 40 × and 200× in overall and partial picture, respectively. Data are presented as mean ± S.D. *n* = 6. *P < 0.05, **P < 0.01 versus the IL-1β group

We used an ex vivo culture model of cartilage explants from nine 4-week-old rats to evaluate effect of UA on cartilage degradation. The cartilage explants were treated with UA (15 μM) with or without IL-1β stimulation (30 ng/ml) for 3 days. The explants were divided into three groups: (1) control, (2) IL-1β stimulated (30 ng/ml), and (3) IL-1β plus (30 ng/ml) UA (15 μM). Histopathological changes in cartilage were evaluated by H&E, S-O Fast green, and Alcian blue staining. As shown in Fig. 6a, h &E and S-O Fast Green staining revealed normal structure of cartilage including smooth and intact surfaces and normal morphology and numbers of well-organized chondrocytes in the control group. However, the IL-1β treated group had apparent morphological changes including rough surfaces (black arrow), clustered and disorganized chondrocytes (black triangle), obvious hypocellularity, and loss of Safranin-O staining compared with the control group. Notably, OARSI scores of the cartilage showed cartilage damage was significantly attenuated by treatment with UA (Fig. 6b). Alcian Blue staining for glycosaminoglycan (GAG) distribution. The control group showed the strongest positive expression of GAG, indicating a sound chondroprotective effect. However, the IL-1β group showed the loss of GAG from the superficial zone to the deep zone of articular cartilage (Fig. 6c). Intriguingly, these changes were slightly decreased by UA. Immunohistochemistry showed that the expression of Collagen II and Aggrecan were reduced by IL-1β (Fig. 6d). Apparently, UA administration could effectively reverse the pathological changes with significantly (Fig. 6, f), which was consistent with the western blot results. Taken together, these results indicate that both the structure and ECM of cartilage tissues were better preserved in the UA-treated group.

## Discussion

OA is traditionally considered a mechanically induced chronic condition and affects more than 25% of the population over 18 years old. Although the occurrence of OA is closely related to multiple factors, the exact pathogenesis remains unclear [27]. Additionally, multiple non-surgical regimens used for OA were limited for many aspects including recurrent side effects and variable rates of success. Thus, a safe and effective drug with a certain molecular target is in urgent need to alleviate cartilage degradation. The gut microbiota and its metabolites could affect multiple organs and contribute to disease progression [28, 29]. Short-chain fatty acids, the main products of intestinal bacterial fermenting dietary fiber, intrigued researchers because of its potential role in the prevention and treatment of metabolic syndrome, bowel disorders, and cancer [30, 31]. UA is a metabolite derived from EA and ETs with a lower molecular weight and better bioavailability compared to its precursors; it is thought to play a protective role in chronic disease with broad spectrum of anti-inflammatory effects. Here, we demonstrated that UA prevented IL-1β-induced damage of ex vivo cartilage explants. Under IL-1β stimulation, UA also attenuated the increased expression of cartilage catabolic enzymes (iNOS, COX2, MMPs) and restored the decreased expression of Sox-9 in rat chondrocytes. Moreover, IL-1β-induced degradation of Collagen II and aggrecan was attenuated by UA. Finally, we found that the MAPK and NF-κB pathways were involved in the protective effects of UA. Collectively, our results suggested that UA may be a promising therapeutic strategy for OA.

Numerous studies have implicated the pro-inflammatory cytokine IL-1β as a vital factor in OA, because it is significantly increased in the synovial fluid of OA patients. Anti-inflammatory treatment plays a key role in alleviating OA symptoms. A recent study demonstrated that the Liraglutide (GLP-1 agonist) ameliorates cartilage degeneration in a rat model of knee osteoarthritis with anti- inflammatory activity [32]. Valproic acid and butyrate were widely reported as latent therapeutic agents for OA due to their ability to suppress IL-1β-induced inflammation and cartilage degradation [33, 34]. During disease progression, IL-1β stimulates the expression of the matrix metalloproteinase (MMPs) that mediate the degradation of cartilage matrix components and suppress proteoglycan synthesis [35]. Our study further revealed that UA obviously inhibited the levels of MMP3 and MMP13 enhanced by IL-1β treatment. IL-1β also promotes the expression of iNOS and COX-2, which induces the production of nitric oxide (NO) and prostaglandin E2 (PGE2), respectively. NO is a well-known inflammatory mediator that can induce MMP secretion and activation and decrease Collagen II and proteoglycan synthesis. In the present study, we found that IL-1β-induced expression of iNOS and COX2 were also attenuated by UA. Sox-9 is a vital transcription factor that positively regulates Collagen II synthesis and is indispensable for chondrocyte differentiation [36]. Our study further pointed out that UA obviously restored the levels of SOX9 inhibited by IL-1β treatment. To sum up, our data showed the therapeutic effect of UA by restoring the imbalance between anabolism and catabolism of the cartilage matrix.

A recent study also found that UA suppressed the excessive production of NO, PGE2, IL-6 and TNF-α in collagenase-isolated human OA chondrocytes [19]. However, chondrocytes isolated from OA patients had showed various phenotypic changes due to destructive changes in the joint which does not seem to reflect the natural process of OA [37, 38]. In contrast, primary chondrocytes, especially cartilage explants in which chondrocytes remain in contact with the extracellular matrix, are more sensitive to molecular environment than OA chondrocytes, thus ex-vivo experiments based on primary chondrocytes and cartilage explants seem to be a more reliable OA-model [38]. In this study, we isolated rat chondrocytes as well as rat articular cartilage explant for experiments, which would provide further evidence that UA may inhibit IL-1β-induced inflammatory response and preserve ECM of cartilage explant tissues.

Various intracellular signaling pathways are reported to participate in OA pathogenesis. The MAPK and NF-κB pathways are master regulators of inflammation and catabolism in the process of OA. MAPK, mitogen-activated protein kinase, is a serine/threonine protein kinase in the cell that transmits extracellular stimulation signals into the cell, causing cellular responses such as cell proliferation, differentiation, transformation, and apoptosis. Under inflammatory stimulation, three major members of this pathway include extracellular regulated kinase (ERK), N-terminal kinase (JNK) and p38 are activated and the phosphorylation levels of them are up-regulated, thus activating the downstream transduction pathway and then activating transcription factors, resulting in the release of pro-inflammatory factors and inflammatory response. For OA, the active form of ERK, JNK and p38 was observed in synovial tissue and cartilage lesion. In addition, several lines of evidence understate that activation of MAPK induced by IL-1β trigger aggrecanases and MMPs-mediated articular cartilage degradation [36, 39]. Specially, Therapy targeting p38 inhibitors could attenuate cartilage degeneration and relief pain in animal models [40].

**Fig. 7:**
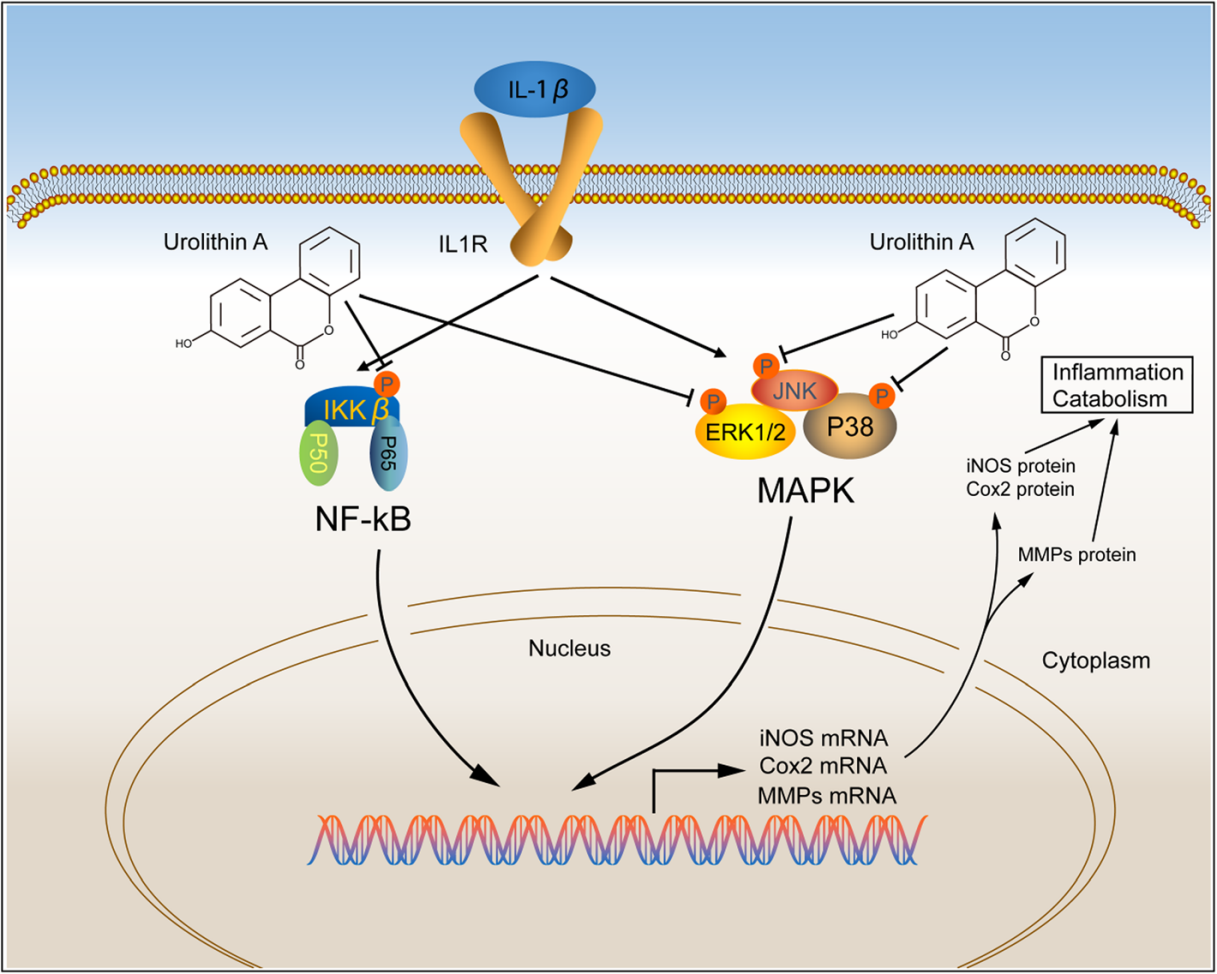
A schematic of the proposed mechanism. During degeneration, MAPK and NF-κB signaling are activated by IL-1β stimulation. UA treatment can decrease iNOS, COX2, and MMPs expression and increase matrix gene expression through inhibiting activation of the MAPK and NF-κB pathways

The NF-κB pathway includes a family of ubiquitously expressed transcription factors and regulates inflammatory responses [17, 41]. Normally, the transcription factor exists in the cytoplasm and is render inactive by a constitutive interaction with the inhibitory protein IκB. Once stimulated by IL-1β, NF-κB p65 translocates into the nucleus where it stimulates the expression of inflammatory mediators such as iNOS, COX-2, and MMPs, among these factors, iNOS catalyzes NO, which stimulates the secretion of MMPs and represses collagen II and proteoglycan synthesis to cause ECM degradation [21, 39]. Previous studies demonstrated that UA could attenuate lipopolysaccharide-induced inflammation by inhibiting activation of the MAPK and NF-κB pathways and alleviate oxidized low-density lipoprotein-induced endothelial dysfunction by modulating MAPK signaling [42, 43]. Our results demonstrated that IL-1β activated ERK, JNK, and p38; upregulated levels of phosphorylated p65; and increased nuclear translocation of p65. In articular cartilage, IL-1β binding to IL-1 receptor results in the recruitment of MyD88, followed by the activation of IRAKs and the E3 ubiquitin ligase TRAF6, and finally activate the MAPK and NF-κB pathway [44]. Furthermore, stimulation of IL-1β induces the accumulation of reactive oxygen species (ROS), known as second messengers during the activation of redox-sensitive transcription factors the MAPK and NF-κB pathway [45]. UA can exert its anti-inflammation and anti-oxidant activity effect through multiple ways. Although its specific target still needs to be explored, our data suggest that UA exerted its beneficial effects by inhibiting MAPK and NF-κB signaling (Fig. 7).

## Conclusions

Our results provide evidence that UA can attenuate IL-1β-induced degradation of Collagen II and aggrecan and reduces the production of inflammatory mediators via the ERK, JNK, P38, and NF-κB pathways in rat chondrocytes. Collectively, these findings suggest that UA may be a promising therapeutic agent in the treatment of OA.

## Data Availability

All data generated or analyzed during this study are included in this published article and are available from the corresponding author upon request.

## Abbreviations

ADAMTs: A disintegrin and metalloproteinase with thrombospondin type 1 motifs
Collagen II: Type II collagen
COX-2: Cyclooxygenase-2
ECM: Extracellular matrix
IL-1β: Interleukin-1β
iNOS: Inducible nitric oxide synthase
MAPK: Mitogen-activated protein kinase
MMPs: Matrix metalloproteinases
NF-κB: Nuclear factor-κB
OA: Osteoarthritis
UA: Urolithin A
